# Properties of V1 Neurons Tuned to Conjunctions of Visual Features: Application of the V1 Saliency Hypothesis to Visual Search behavior

**DOI:** 10.1371/journal.pone.0036223

**Published:** 2012-06-12

**Authors:** Li Zhaoping, Li Zhe

**Affiliations:** 1 Department of Computer Science, University College London, London, United Kingdom; 2 School of Medicine, Tsinghua University, Beijing, China; Indiana University, United States of America

## Abstract

From a computational theory of V1, we formulate an optimization problem to investigate neural properties in the primary visual cortex (V1) from human reaction times (RTs) in visual search. The theory is the V1 saliency hypothesis that the bottom-up saliency of any visual location is represented by the highest V1 response to it relative to the background responses. The neural properties probed are those associated with the less known V1 neurons tuned simultaneously or conjunctively in two feature dimensions. The visual search is to find a target bar unique in color (C), orientation (O), motion direction (M), or redundantly in combinations of these features (e.g., CO, MO, or CM) among uniform background bars. A feature singleton target is salient because its evoked V1 response largely escapes the iso-feature suppression on responses to the background bars. The responses of the conjunctively tuned cells are manifested in the shortening of the RT for a redundant feature target (e.g., a CO target) from that predicted by a race between the RTs for the two corresponding single feature targets (e.g., C and O targets). Our investigation enables the following testable predictions. Contextual suppression on the response of a CO-tuned or MO-tuned conjunctive cell is weaker when the contextual inputs differ from the direct inputs in both feature dimensions, rather than just one. Additionally, CO-tuned cells and MO-tuned cells are often more active than the single feature tuned cells in response to the redundant feature targets, and this occurs more frequently for the MO-tuned cells such that the MO-tuned cells are no less likely than either the M-tuned or O-tuned neurons to be the most responsive neuron to dictate saliency for an MO target.

## Introduction

### Background on visual attention, saliency, and their neural substrates

Spatial visual selection, often called spatial attentional selection, enables vision to select a visual location for detailed processing using limited cognitive resources [1]. It can be generated by goal-dependent (or top-down) mechanisms, such as when we direct our gaze to a book while reading, or by goal-independent (or bottom-up) mechanisms such as when we are distracted from reading by a sudden appearance of something in visual periphery. In this paper, an input is said to be salient when it strongly attracts attention by bottom-up mechanisms, and the degree of this attraction is defined as saliency. Saliency of a visual location is often measured by the speed of a visual search to find a target at this location [2], or by its attentional (exogenous) cueing effect (i.e., the degree it speeds up and/or improves visual discrimination of a probe presented immediately after the brief appearance of the salient cue) [3], [4].

It has been proposed that the primary visual cortex (V1) is responsible for computing saliency [5], [6]. Although this V1 saliency hypothesis is a significant departure from traditional psychological theories [2], [7], [8], [1], in which the neural substrates are not their main concern, it has received substantial support [9]–[15]. In particular, behavioral data confirmed an unexpected prediction that an eye of origin singleton (e.g., an item uniquely shown to the left eye among other items shown to the right eye) that is hardly distinctive from other visual inputs can attract attention and gaze qualitatively just like a salient and highly distinctive orientation singleton does – in fact observations [13], [15] show that the eye of origin singleton can be more salient than an orientation singleton. This finding provides a hallmark of the saliency map in V1, because the eye of origin feature is not explicitly represented in any visual cortical area except V1. Functional magnetic resonance imaging and event related potential measurements also confirmed that, when top-down confounds are avoided, a salient location evokes brain activations in V1 but not in the parietal and frontal regions [14], which are thought to be involved in saliency by traditional views [1].

**Figure 1:**
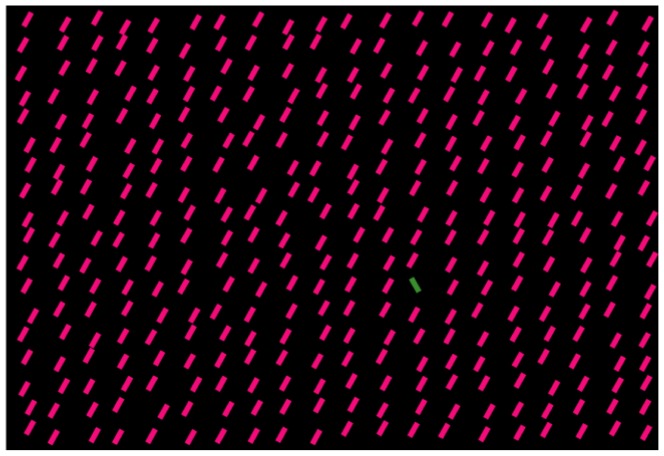
A schematic example of the search stimulus by Koene and Zhaoping [10]. Data in their behavioral study are used for the current study. Observers searched for a bar unique in color (C), orientation (O), or motion direction (M), or a combination of these features. In this illustration, the target is a double feature CO target, unique in both color and orientation. See Method or the original paper [10] for the actual stimulus details.

In another study, Koene and Zhaoping [10] measured RTs for finding a target bar unique in color (C), orientation (O), motion direction (M), or redundantly in two of these features (CO, MO, or CM) among background bars which are identical to each other in all features (see Fig. 1). If the RT for a redundant target (e.g., a CO target) is the shorter one of the two RTs for the corresponding single feature targets (e.g., the C and O targets), the RT for the redundant target is said to be the outcome of a race model between the two other RTs (as if to take the RT of the winner in a race between two racers) [16]–[19]. Since RTs are stochastic, the RT from a race model is also said to be the result of a statistical facilitation between the RTs of the individual racers. If the RT for a redundant target is shorter than predicted from the statistical facilitation, there is a redundancy gain [20]. According to the V1 saliency hypothesis, the presence or absence of the redundancy gain in behavior should reflect the presence or absence, respectively, of V1 neurons tuned simultaneously to the two visual features distinguishing the redundant target. We call such cells conjunctively tuned CO, MO, or CM cells, each denoted by the feature dimensions in which they are tuned. Koene and Zhaoping [10] found this redundancy gain for the CO and MO targets but not the CM target, supporting the V1 saliency hypothesis since V1 has CO and MO cells [21], [22], [23], but no CM cells [24].

The finding by Koene and Zhaoping [10] also implies that the extrastriate cortices are unnecessary for the bottom-up saliency of their singleton targets. This is because extrastriate cortices do have the CM conjunctive cells [25], [26], which would have led to a redundancy gain in the CM targets. The implication is consistent with another behavioral observation involving depth cues, which are believed to be processed in extrastriate but not V1 [27]–[32]. It was found that depth cue did not speed up attentional guidance to a target location unless this location was not salient enough to be reported by observers within an RT of one second [33], which is about twice as long as typical RTs to report a feature singleton in Koene and Zhaoping's study. Longer RT events are likely to involve top-down and object/surface recognition processes beyond the bottom-up saliency process (which dominates only in short RT events [34]), and involve extensive neural connections between V1 and extrastriate cortices [35], [36], [37]. In addition, the findings by Koene and Zhaoping [10] and others [11] are consistent with the feature combination rule to compute saliency according to the V1 saliency hypothesis. According to this rule, saliency at a location is determined by the highest V1 neural response to that location, without combining responses from multiple neurons responding simultaneously to different input features at the same location. In contrast, the feature combination rules by the traditional saliency models (reviewed by Itti and Koch [1]) compute the saliency value at a location by summing responses to this location from various basic feature maps. Apparently, V1 does not perform any summation across feature dimensions. Hence, higher cortical areas have to be involved if feature summation is to occur for computing a saliency map.

### The goal and the plan for the current study

**Figure 2:**
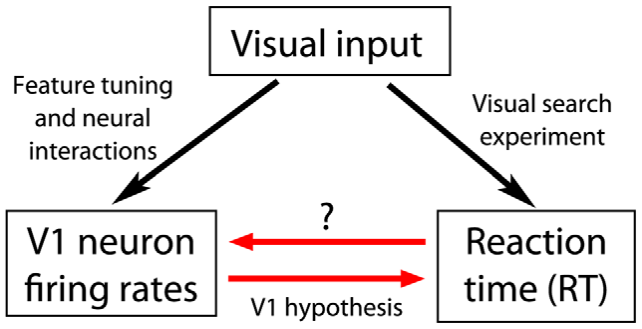
The schematic of our method to probe V1 properties through behavior. Visual inputs drive V1 responses. Meanwhile, the V1 responses determine the behavioral RTs in visual search tasks, according to the hypothesis that the V1 responses represent saliencies of input locations. Therefore, one may probe V1 properties through the relationship between the RTs and V1 responses. In particular, a shorter RT arises from a higher V1 response to the search target relative to those to the background items. Therefore, from the RT data, one can infer relative response levels of the V1 neurons, thereby probing the feature tuning of the V1 neurons and interactions between the neurons.

Whereas the previous studies used known facts about V1 physiology to test, and confirm, the V1 saliency hypothesis, the current study aims to probe the unknown or less known V1 properties assuming that the V1 saliency hypothesis holds (Fig. 2). In particular, Koene and Zhaoping [10] confirmed that the V1 saliency hypothesis is supported by the known facts that V1 contains CO and MO cells but no CM cells. Meanwhile, many physiological properties associated with these conjunctive neurons are less known, or have not been systematically studied. In particular, one would like to ask the following questions. How responsive these conjunctive neurons are compared to the other neurons? How do the intra-cortical interactions between these neurons vary with the feature preferences of the interacting neurons? The current study uses the V1 saliency hypothesis to investigate these less known properties from the behavioral RT data collected by Koene and Zhaoping [10]. To do so, we formulate a computational approach based on the V1 saliency hypothesis to solve for aspects of the V1 neural properties from the behavioral RT.

For this study, the theoretical basis is the V1 saliency hypothesis. The hypothesis states that the saliency of a visual location is represented by the highest V1 response to this location relative to the background responses [5], [6]. This is regardless of whether this response is from a neuron tuned to orientation (O), color (C), motion (M) direction [38], [39], or other features, or conjunctively to two feature dimensions (e.g. CO or MO) [38], [39], [21], [22], [23]. In particular, according to this hypothesis, the most salient location in a scene is the receptive field (RF) of the most activated V1 neuron responding to this scene, regardless of the preferred feature(s) of this neuron. A feature singleton, such as the search target in Koene and Zhaoping [10] (see Fig. 1), can evoke the highest response to the scene because of a neural property called iso-feature suppression [40]. Iso-feature suppression means that V1 neurons tuned to the same or similar features tend to suppress each other's responses via intra-cortical neural connections when their RFs are close to each other [41], [42], [43]. For example, a unique vertical bar is very salient in a background of horizontal bars, since different neurons (preferring horizontal orientation) responding to different and neighboring horizontal bars suppress each other by iso-orientation suppression [42], while the neuron (preferring vertical orientation) responding to the unique vertical bar escapes such suppression. Iso-color [44] and iso-motion-direction [45] suppressions are other known examples of iso-feature suppression. To make the highest response to the feature singleton target sufficiently higher than those to the background bars, the following two conditions are required. First, the intra-cortical interactions are sufficiently feature specific such that iso-feature suppression is only substantial between two neurons whose preferred feature(s) are sufficiently similar. (In principle, it should also work if the iso-feature suppression is much stronger when the two neurons prefer sufficiently similar feature(s) than otherwise.) Second, the input feature preference of the neurons should sufficiently differentiate the target and background features. We call these two elements *feature tuning of intra-cortical interactions* and *feature tuning of individual neurons* respectively. Sometimes, *feature tuning of individual neurons* is also referred to as *feature tuning of input preferences*.

Usually the feature singleton search target in Koene and Zhaoping [10] evokes responses from many cells tuned to different features. Some of these cells are tuned to color (C), orientation (O), or motion direction (M), and some are tuned to conjunctions (e.g., CO, MO) of them. We call a neuron a C, O, or M neuron if it is tuned in a single corresponding feature dimension, and a CO, MO, or CM neuron if it is tuned conjunctively in the two corresponding feature dimensions. According to the V1 saliency hypothesis, the highest response among the responses (to the target) from all neurons determines the saliency of the target. This saliency in turn determines the RT to find the target. For example, for a color singleton, a C cell's response is expected or assumed (see Discussion) as most likely to dictate its saliency. Meanwhile, for a CO singleton target in Fig. 1, the dictating response could come from a C, O, or a CO cell, depending on the feature tunings of these cells and of the intra-cortical interactions. We will show that some aspects of these neural properties can be revealed from the RT data through the solution of an optimization problem formulated from the V1 saliency hypothesis.

Previous works [5], [6], [40], [46], [47] have introduced a V1 model to simulate and analyze the intra-cortical mechanisms in order to understand the neural mechanisms behind the V1 saliency hypothesis. We like to point out that the current work probes the V1 neural properties using the V1 saliency hypothesis, the theory, rather than this V1 model. The theory presents a hypothesis about the functional role of the V1 responses, and states that the intra-cortical mechanisms serve to highlight V1 responses to conspicuous locations where input statistics deviates from translation invariance [5], [6], [40]. In contrast, by simulating the mechanisms in V1 that give rise to these responses, the model tests whether it is feasible that V1 responses might play the hypothesized role. For simplicity, this V1 model, or model V1, has so far included only model neurons tuned to orientation, except in two examples in which model neurons tuned to color or color-orientation conjunctions are also included [6], [9]. However, the theoretical hypothesis is general regarding input feature dimensions and neural mechanisms as it refers to the real, physiological, V1, rather than the simplistic and inaccurate model V1. Indeed, various behavioral tests of the hypothesis have included both the modeled and not modeled feature dimensions: orientation, color, motion direction, and ocular origin [9]–[14], since the model V1 is unnecessary when the physiological V1 in human observers are available for these behavioral experiments. Similarly, our formulation, method, and results in the current study depend only on the V1 saliency hypothesis and the general knowledge about the physiological V1, and not on the model.

Our predicted V1 properties from applying the V1 saliency hypothesis to the behavioral data can serve two purposes. First, they can motivate physiological experiments to test the predictions, thus providing further test of the V1 saliency hypothesis. Second, they enable the use of a computational theory as a tool to investigate physiological properties from behavioral data without physiological experiments. We will discuss the implication of our findings in the Discussion.

## Methods

### Behavioral data

The RT data are collected by Koene and Zhaoping [10], which contained all experimental details. In that study, verbal consents from all participants were obtained, as documented by the subject information in the data. The study and the consent procedure were approved by the ethics committee in University College London. Briefly, the search display contained an array of 30
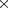
22 colored, tilted, and moving bars. Observers were instructed to find the target bar as soon as possible, and their RTs to find it were measured. There were only two possible iso-luminant colors (green or purple of the same saturation), orientations (left or right tilted from vertical by the same angle), and motion directions (moving to the left or right at the same speed) for all stimulus bars in any search trial. All non-target bars were identical to each other in color, orientation, and motion direction (see Fig. 1), and the target differed from the non-target bars in color, orientation, motion direction, or redundantly in more than one feature dimension. In each search trial, the choices of the target and non-target features were random, and the choice of feature dimension(s) in which the target differed from the non-target was also random. Hence, the possible target conditions included C, O, M, CO, MO, and CM, each defined by the feature dimensions in which the target feature was unique. Each bar was about 1 degree long and 0.2 degree wide. The positions of the bars were randomly jittered from their regular grid locations, such that the horizontal distance between neighboring bars ranged between 1.2 to 3.3 degree and the vertical distance between them ranged between 1.1 to 2.0 degrees. The data considered in this study are from the search trials in which the target bar was at a random location roughly 12.8 degrees from the display center, and at least 11 degrees horizontally. The observers were instructed to press a left or right button as soon as possible for a target (present in each trial) in the left or right half of the stimulus array, respectively. For a given target condition and a given subject, the mean and standard deviation of the RTs for the correctly performed trials were obtained, and RT outliers are defined as those shorter than 0.2 second or longer than 3 standard deviations from the mean. RTs included for this study exclude the RT outliers and those in trials with an incorrect button press. When the target was unique in color, orientation, or motion direction only, it is called a single feature target; when a target was unique in two features, it is called a double or redundant feature target. For each subject, the orientation and color difference between the target and non-targets, and the motion speed, were roughly pre-adjusted, such that the subject had a mean RT of about 600 ms for each single feature target type. Typically, the average RTs for the double feature targets were around 500 ms as a consequence. Each subject did about 320 search trials for each target type. The percentage of trials excluded in our data analysis, due to button press errors or to the RT being an outlier, is no more than 9.2% (about 5% in average) for each subject in each condition. There were eight subjects, including the authors, Koene and Zhaoping, and six naive subjects.

### Extracting properties of the conjunctive cells from behavioral data

For simplicity, we will often narrate as if there is only one cell of each cell type responding to each visual location (or bar) in a search stimulus. This one cell should be understood as the most activated cell of the given cell type. This is because, as far as saliency is concerned, the less activated cells by inputs at a given visual location are irrelevant according to the V1 saliency hypothesis. For the same reason, we often omit an entire cell type when considering neurons and their responses to a visual location, as long as the omitted cells are not the most responsive. For example, to a color singleton target among non-target bars, which have the same orientation as the target bar, the dominating responses are most likely from the C cells rather than the O cells, which are suppressed by iso-orientation suppression. In such a case, the analysis will often omit mentioning the O cells at all.

#### Linking V1 responses with search RT

Due to iso-feature suppression, the most activated V1 neuron to the search stimuli is most likely the ones responding to the target. For example, a C neuron preferring green will respond most vigorously to a green singleton target among purple distractors. Meanwhile, the population responses to non-targets should be approximately those evoked by a stimulus identical to the search stimulus except for replacing the target by a non-target bar. The level of this population response pattern should be independent of whether this uniform group of bars are green or purple (of the same luminance and saturation), left or right tilted (by the same angle from vertical), and moving to the left or right (at the same speed). Therefore, we make the approximation to view the level of the population responses to the non-targets as independent of the target conditions. Consequently, within the class of the search stimuli in our analysis, the target's saliency is a monotonic function of the highest response 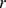 evoked by the target. Since a more salient target leads to a shorter RT by definition, a higher response 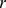 maps monotonically to a shorter RT by a mapping 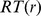, which depends on the mechanisms of saliency read-out and ocular-motor functions. For example, let 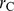 be the highest response from the C cells to the color singleton target. Given an observed 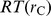 to find the target, one can infer the unobserved neural response 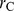 if the mapping 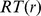 is known. Stochastic nature of the neural system gives a distribution of 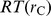 from many search trials, arising from a corresponding distribution of 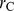
's.

#### Obtaining properties of conjunctive cells from the RTs using a race model

Throughout the rest of the Method section, we often use a CO target as an example to derive and illustrate how to probe neural properties, e.g., the relative levels of neural responses 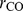, associated with conjunctively tuned cells. The methods and arguments apply analogously to the cases of other double feature targets and neurons.

**Figure 3:**
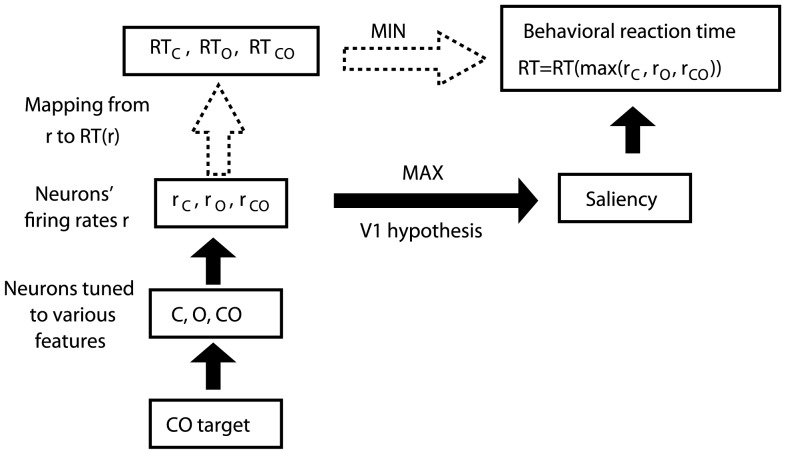
A schematic of the relationship between V1 responses and search RTs. In this example, a CO target activates three types of V1 neurons, tuned to C, O, and CO respectively. Their responses, 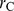, 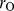, and 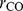 , are influenced by intra-cortical mechanisms in V1. Their maximum 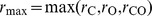 determines the target's saliency. Thus the behavioral RT is a function of 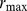 (with distractor responses normalized to 1), through a monotonically decreasing mapping 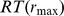 determined by the brain mechanisms for saliency read-out and ocular-motor functions. Equivalently, the behavioral RT is 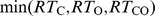, as the result of a race between the racers C, O, and CO, whose RTs are, respectively, 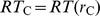, 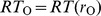, and 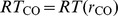.

**Figure 4:**
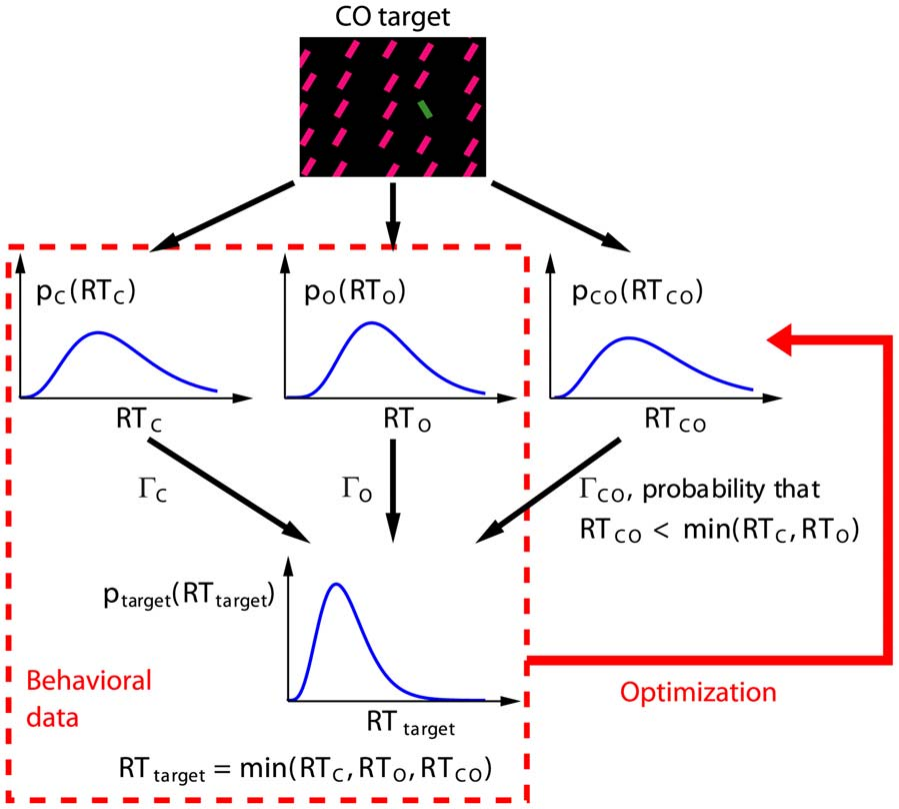
Deriving contributions by various V1 neurons to a double feature target's saliency from the RT. This is illustrated by the example of a CO double feature target. The stochastic V1 responses 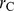, 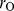, and 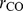 lead to stochastic 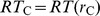, 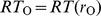, and 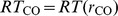, with probability distributions 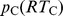, 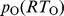, and 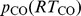 respectively. The C, O, or CO cell is the winner of the race with probability 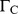, 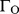, and 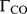 respectively, giving target 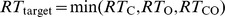. Samples from the probability distributions 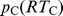, 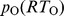, and 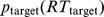 are measured as the behavioral RT data for targets C, O, and CO, respectively. From these data, the underlying probability distributions 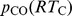, 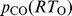, and 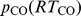 can be inferred by an optimization procedure, and the three contributions 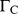, 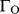, and 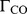 can then be calculated.

The saliency for a CO target is not necessarily dictated by 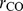, the response from a CO cell, but by the maximum of 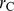, 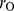, and 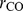, the responses from the C, O, and CO cells, respectively. In other words, the RT for the target is 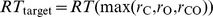, where max(…)denotes the maximum of the arguments. This method to obtain the RT for a double feature target is called a race model [16]–[20], which intuitively assigns as the RT for the target the winning RT in a race between three racers whose respective RTs are 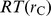, 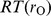, and 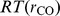 (Fig. 3, 4). For notational convenience, 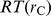, 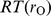, and 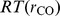 are also denoted as 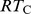, 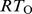, and 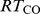, respectively. Therefore (Fig. 3),
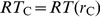
(1)

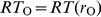
(2)

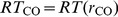
(3)

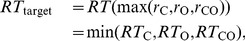
(4)where min(…)denotes the minimum of the arguments.

The neural activities 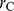 (or 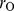) are assumed to follow the same probability distribution whether the target is a single feature C (or O) target or a double feature CO target. Hence, the probability distributions of 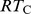, 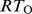, and 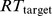 are sampled by behavioral RT data from C, O, and CO target trials respectively. Additionally, 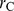, 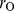, and 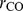 are assumed to be randomly and independently drawn from their respective distributions. Consequently, 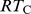 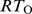 and 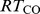 are also randomly and independently drawn from their respective distributions. Meanwhile, 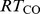, which cannot be measured behaviorally, can be inferred from other behavioral data. For example, if a 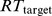 sample is shorter than all samples of 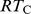 and 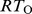, it is likely to represent an underlying 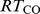 sample according to equation (4). More generally, even when a 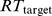 is not shorter than all samples of 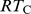 and 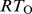, it is still possible to represent a 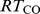 sample if its occurrence is more likely than expected from random races between only two racers with 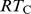 and 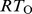 respectively. More formally, an optimization method (see a later section on technical details) can be used to infer the underlying distributions of 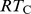 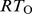 and 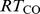 from the behavioral RT samples. Since a monotonic function relates 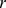 and RT, relative activity levels among 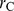, 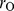, and 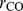 can then be inferred from the relative values among 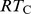 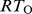 and 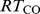, even though the exact form of the mapping from 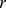 to 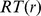 is not known and is subject dependent.

#### Obtain the impacts or contributions of different cells in visual search

The contribution of a neuron to the saliency of a double feature target can be obtained even if the neural activities are not absolutely known. For a CO target, for example, the contribution of the CO cells to the target's saliency is defined as the probability that the CO cell gives the highest evoked response (or, equivalently, wins the race among the three racers), i.e.,
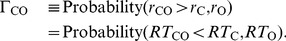
(5)Similarly, the contributions from the C and O cells are, respectively,
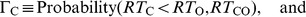
(6)

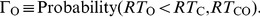
(7)


In our data analysis, probability distributions of the RTs are described by probabilities of the RTs in discrete time bins. Due to the finite sizes of these time bins, there is a non-zero probability that more than one racer jointly win a race (by being in the same bin), giving 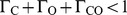. However, this does not change our qualitative conclusions.

One can easily imagine that if the mean 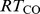 is substantially longer than those of 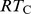 and 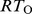, the contribution 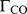 by the conjunctive CO cell will be likely close to zero. In contrast, if contribution 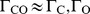, the responses by the conjunctive cells are comparable to those by the single feature tuned neurons.

#### Assessing the significance of the roles of the conjunctive cells

By definition, the 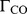 will never be negative. Meanwhile, the finite numbers of behavioral samples in our data imply that our sampled probabilities 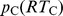, 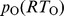, and 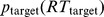 are noisy versions of the actual probabilities. Consequently, a positive contribution 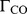 is likely obtained even if 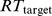 were sampled from the race between 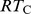 and 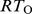 only. We define 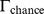 as the chance level 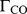 value obtained by replacing the 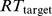 data by as many trials (as the number of the CO target trials) of this simulated race winner 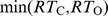 between the two racers using Monte Carlo method [10]. We obtained 1000 evaluations of 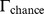, each from a random set of sampled RTs of the race winner. 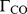 is said to be significant if it is larger than 95% of these 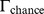 values, i.e., 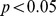.

### The verification of consistency and validity of our method

**Figure 5:**
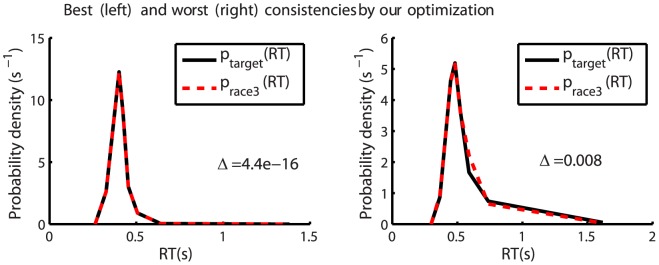
Visualization of the consistency of our method. Shown are the best and the worst consistencies in using our optimization method to probe the double feature tuned cells, among all subjects and all double feature target conditions. A better consistency means a better match between the two curves 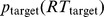 and 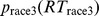. Here, 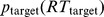 is the distribution of the behavioral RT data for a double feature target. Meanwhile, 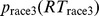 is the corresponding distribution of the winning RT from a race between the three RT racers (e.g., see Fig. 4) whose probability distributions are inferred from the behavioral RT data by our optimization method. In most cases (not shown here), the two curves are not visually distinguishable, similar to that in the plot for the best case.

To verify the consistency of our method, we checked after optimization whether 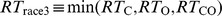, the race winner among the three racers C, O, and CO, has the same distribution as that of our behavioral 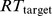 for the CO target. A large difference between these two distributions indicates a poor performance of our optimization method, and consequently, unreliable results and conclusions from the method. This consistency can be quantified by 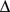 (defined as 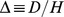, where 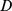 is the K-L divergence between 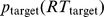 and 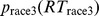, 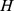 is the entropy of 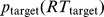, and both RT distributions are discretized by the same time bins for the calculation), such that a 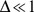 indicates a good agreement between the two distributions. Fig. 5 shows the best and worst consistency cases. For all subjects and double feature target conditions, 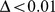, and typically the curves of 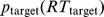 and 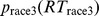 are not visually distinguishable. An analogous 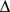 value can also be calculated for the probability distributions for any given single feature target, when 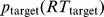 and 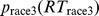, respectively, are replaced by the measured and inferred (by the optimization) probability distributions of a given singleton target. For all subjects and all single feature targets, such 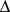 values are all smaller than 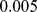. Hence, our optimization method is highly reliable and gives consistent results.

Meanwhile, our calculated contributions 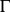 by the various feature tuned cells depend on the number and the placement of the time bins to discretize the RT data. Smaller bins give fewer RT samples in each bin, making the sampled distributions noisier and 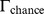 larger. Larger bins give coarser distributions, making it more difficult to distinguish the race winner, since joint winners in a race are more likely. Given the number 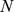 of the bins, we place the bins such that each of the first 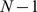 bins contains roughly the same total number of RT samples from all target types, while the last bin is the reserve for possible long RTs (from the double feature cells) which never wins the race. Given our RT data, when 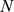 is between 7–13, such that the probability that the race is won by joint winners is on average between 10–20%, our results do not qualitatively depend on the number 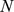 of the bins. This paper shows the results for when 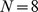.

### Technical details in the methods

#### Optimization method to calculate RT distributions generated by the responses of various types of neurons

For each subject and each target type, the RTs in the 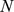 time bins are described by a vector 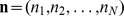, with 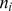 = the number of RT samples in the 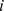
th time bin with 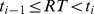. Let 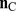, 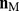, 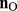, 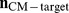, 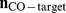, and 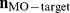 denote these vectors for targets C, M, O, CM-target, CO-target, and MO-target respectively for a given subject. Let the probability distributions 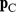, 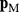, 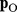, 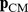, 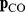, and 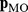 denote the probability of 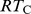, 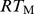, 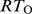, 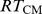, 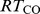 and 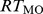 respectively in these same time bins. Their likelihood given 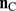, 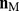, 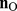, 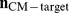, 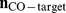, and 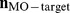 is
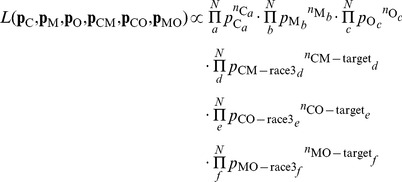
(8)In the above equation, 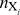 and 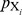 denote the 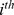 element in the vector 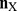 and 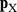 respectively, for X = C, M, O, CM-target, CO-target, MO-target, CM-race3, CO-race3, or MO-race3. Meanwhile, 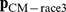 is the probability distribution of the RTs as the result of a race between three racers whose RTs follow probability distributions 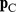, 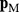, and 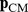 respectively, i.e.,
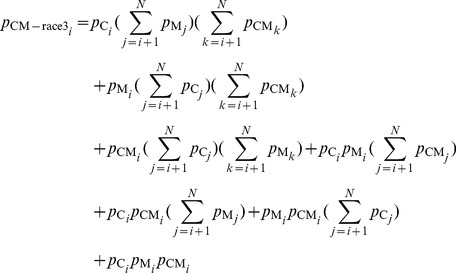
(9)Similarly, the components of 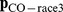 and 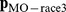 are
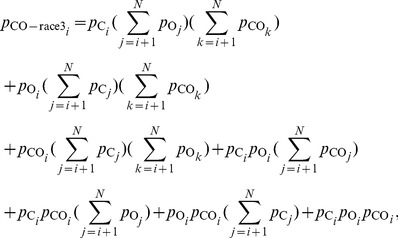
(10)

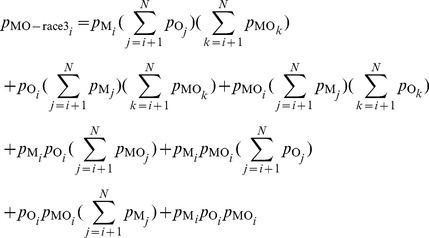
(11)We obtain the most likely 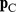, 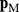, 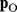, 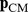, 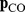, and 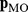 by minimizing the negative log likelihood 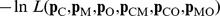. For this optimization, we use the “fmincon” function in MATLAB, imposing the constraint that each of 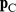, 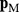, 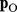, 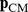, 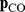, and 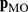 has non-negative components and is normalized, e.g., 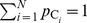.

#### Quantifying the consistency of our optimization method

To quantify the consistency of our optimization method, we first obtain an unbiased estimation of 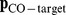 of the 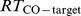 as
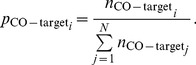
(12)The difference between 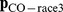 (as in equation (10) above) and 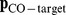 can be measured by Kullback-Leibler divergence
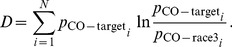
(13)The quality of the consistency of our optimization is quantified by 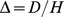, where 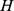 is the entropy of 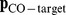


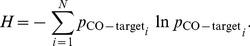
(14)


#### Calculating the contributions by various cell types to the saliency of double feature targets

For example, for the CO target, the contributions 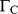, 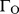, and 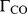 are respectively
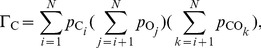
(15)

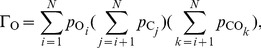
(16)

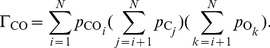
(17)The contributions in the case of other double feature targets are obtained analogously.

#### The policy of placing the time bins

Let 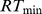 and 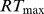 be, respectively, the minimum and maximum RTs of a subject regardless of target types. Let 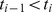 be the boundaries of the 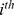 time bin containing RTs satisfying 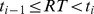. Given the number 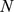 of time bins, the boundaries 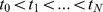 are chosen such that *t*_0_ = *RT*_min_−0.0001 second, *t*_N-1_ = *RT*_max_+0.0001 second, *t*_N_ = ∞, and, if 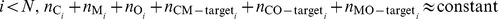 which does not depend on *i.*The last time bin bounded by 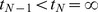 serves as a reservoir for the possibility of the long *RT*_CM,_ *RT*_CO,_ and *RT*_MO_ which never win the races and thus could not be manifested in (or determined by) the behavioral RT data.

## Results

**Figure 6:**
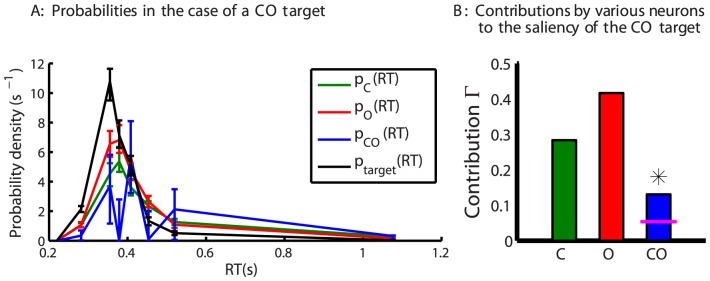
The results for the CO target from a typical subject. A: probability densities for 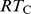, 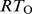, 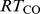, and 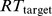. Each density function is plotted as piece-wise lines linking discrete points, with the 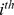 point at 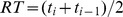 horizontally and 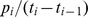 vertically, where 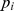 is the probability that the corresponding RT is in the 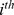 time bin (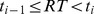). All curves start at 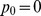 at 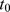. For 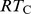, 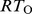, and 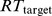, the probability 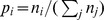, where 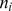 is the number of RT samples in the 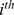 time bin for the corresponding target. For 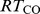, 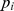 is from the outcome of the optimization. The error bars are generated as follows. For each target type, let 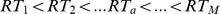 be all the behavioral RT samples included, and let the cumulative RT distribution 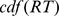 for this target be approximated by a function which has piece-wise interpolations between discrete functional values 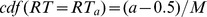 and has 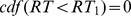 and 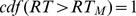. Randomly generate 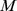 simulated RT samples using this 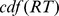. Using these simulated RT samples (as if they were the original RT data) for all target types, we obtain another measurement of the probability densities for all target types and, via our optimization method, all neuron types. Repeat such measurements 500 times. Each error bar has its lower and higher values at the 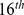 and 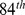 percentiles, respectively, of the corresponding density measurements. B: Contributions 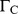, 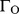, and 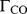 of the C, O, and CO neurons, respectively, to the saliency of the CO double feature target for this subject. Each contribution is the probability that the corresponding neuron dictates the saliency of the CO target (by giving the highest response among responses from all three types of neurons to the CO target). In obtaining their values, probabilities of 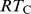 and 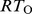 from optimization outcomes, rather than behavioral data, were used. The ‘*’ on top of 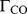 indicates that 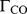 is significantly different from 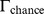 whose mean value is marked by the magenta line.

For the CO target, Fig. 6A shows the probabilities of 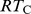, 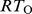, 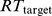 from the behavioral data and that of the inferred 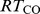 by the optimization for a typical subject. As expected for the RTs of the race winner, the 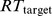 is generally smaller than all the other RTs (of the individual racers). Fig. 6B shows that, for this subject, the saliency of the CO target is determined most likely by the V1 neuron tuned to the O feature, with 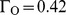, and least likely by the neuron tuned to the CO feature, with 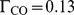. However, 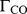 is significantly larger than 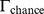 which is typically around 0.05 for all subjects and double feature target conditions. Hence, for this subject, the CO neuron is so responsive to the CO target that it has a substantial probability of 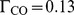 to respond more vigorously than the single feature tuned C and O cells to the CO target.

In Fig. 6A, the distribution of the inferred 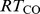 is multi-modal, unlike typical RT distributions. This does not mean that our optimization is faulty, as it is caused by the following. First, the race model is better at determining the shorter 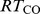
s which are more likely to win the race to be manifested as 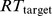. The longer 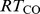
s are under-determined and are largely determined by the probability normalization constraint. Meanwhile, these longer 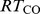
s matter little to 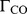 since they do not win the race. In fact, Fig. 6A omitted the last time bin, which contains no behavioral RT samples for any target types but absorbs the longer 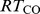
s which never even jointly win the race. Second, our RT data do not allow us to determine how likely it is that the CO cell is the most activated neuron by the C or O target to dictate 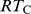 or 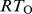, respectively. We have thus for simplicity assumed that the CO cells never dictate 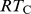 or 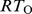 for the C or O targets respectively. If, however, CO cells do dictate 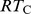 and 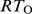 occasionally, the RTs by the C and O neural racers should be longer than those shown in Fig. 6A, and, consequently, some more trials of 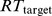 should be attributed to 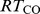 to make 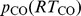 resemble typical RT distributions. The analysis above implies that our inferred 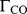 is in fact the additional contribution by the CO neurons beyond their hidden contribution which has been attributed to the C and O cells for simplicity (see Discussion for more details). Third, the probabilities inferred from finite numbers of RT samples are noisy, contributing to the irregularity in the inferred 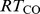 distribution.

**Figure 7:**
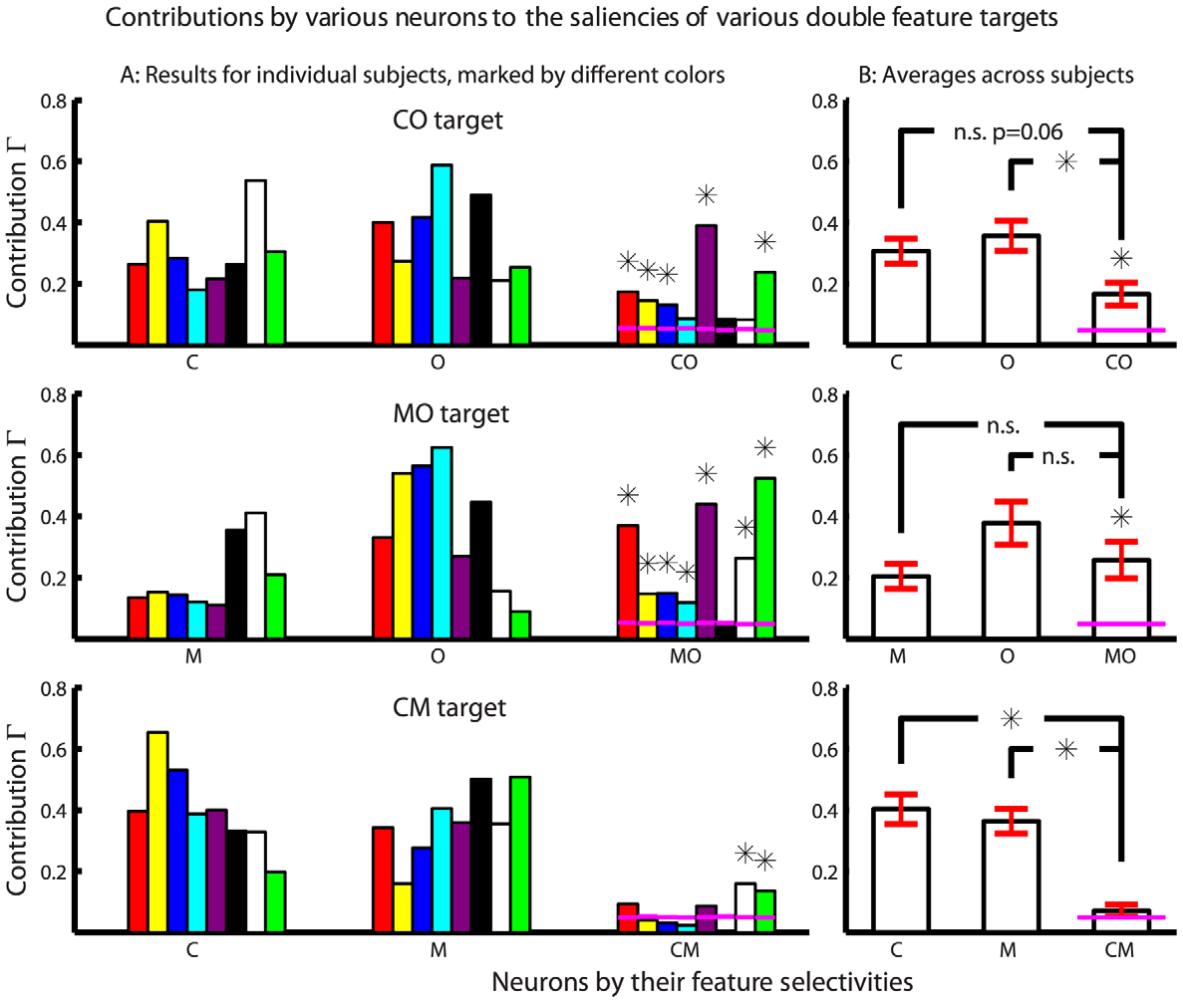
Contributions by various feature-tuned neurons to the three types of double feature targets. Results are shown for individual subjects in A and averaged across subjects in B. The two subjects marked by white and green colored bars are Koene and Zhaoping, experimenters for the behavioral data and the only non-naive subjects. The plots are in the same format as that in Fig. 6B. In B, 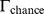 is averaged across subjects, and a subject-averaged contribution by the conjunctive cells is marked as significant if it is significantly different (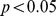) from this 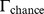 by a t-test. The error bars mark the standard errors of the means. An ‘*’ above a bar for the double feature tuned cell (in A or B) indicates that the contribution by this cell (to the saliency of the double feature target) is significantly above the chance level. In B, an ‘*’ or ‘n.s.’ linking the contribution of a conjunctive cell and that of a single feature tuned cell marks, respectively, a significant or insignificant difference between them (by a matched sample t-test). Qualitatively the same results are obtained in B when data from non-naive subjects, Koene and Zhaoping, are excluded.

Fig. 7 shows the contributions by various feature tuned neurons to the saliencies of different double feature targets for all subjects. Fig. 7A shows that, among 8 subjects, 5, 7, and 2 subjects have their conjunctive cells contributing significantly to the corresponding double feature targets CO, MO, and CM respectively. A t-test is used to see whether the subject-averaged contribution by any double feature neuron is significantly larger than the subject averaged chance level 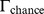. The answer is affirmative except for the CM cells, confirming the conclusion by Koene and Zhaoping [10] that the behavioral RTs for a double feature CO target or MO target, but not the CM target, is significantly shorter than predicted from a race between the RTs for the two corresponding single feature targets. In addition, the current results reveal quantitatively the impacts of the double feature tuned neurons to the saliencies of the double feature targets, and compare them with the impacts of the single feature tuned neurons. Averaged across subjects, 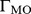 is not significantly different from 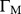 and 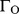, but 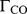 is significantly lower than 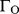 and marginally lower than 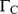. Hence, the MO cells have a larger impact than the CO cells on the saliency of their corresponding double feature target. In particular, the chance 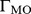 for the MO cell to be the highest responding neuron to dictate the saliency of a MO double feature target is no less than that (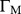 or 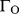) for either of the single feature tuned M and O cells. Meanwhile, the chance 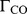 for the CO cell to be the highest responding neuron to dictate the saliency of a CO double feature target is substantial, but is only about half of that (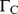 or 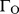) for either of the single feature tuned C and O cells. These results will be used to infer the less known properties of the double feature cells in Discussion.

Note that the relative contributions 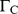, 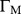, and 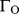 in our results cannot be generally interpreted as relative significance of the roles played by the corresponding single feature tuned cells. For example, if a much smaller orientation contrast between a target and non-targets were employed in our stimuli for O, CO, and MO targets (note that the same orientation contrast was used in these targets by our experimental design), then the saliency of the CO target would be due more to its unique color rather than its unique orientation, and, similarly, the saliency of the MO target would be due more to its unique motion direction rather than its unique orientation. Consequently, the ratio 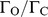 for the CO target and the ratio 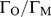 for the MO target will be reduced. Nevertheless, our conclusions regarding the contributions by the conjunctive cells relative to those by the single feature tuned cells should not be as sensitive to the exact feature contrasts in the stimuli, since the conjunctive cells have to be more active than both of the corresponding single feature cells to make an impact.

## Discussion

### Summary of the results and their predictions on V1 physiology

Using RTs in visual search for feature singletons to assess the saliencies of the search targets, and using the V1 saliency hypothesis, this study probes the properties of the less-known V1 cells tuned conjunctively to more than one feature dimension. We are particularly interested in the activities of the conjunctively tund neurons relative to those of the single feature tuned neurons. These relative activities, when they are sufficiently high, make their impacts on the saliencies of the visual inputs, such that they can shorten the RT to find a double or redundant feature target beyond that predicted by a statistical facilitation between the two corresponding single feature targets. In other words, the relatively higher activities of the conjunctive neurons can be manifested as redundancy gains in the RTs of the double feature targets [20]. The relative activities of the conjunctive neurons can be quantified from the redundancy gains by applying the V1 saliency hypothesis. The results show that (1) the chance 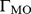 for the MO cell to be the most active neuron in response to a MO double feature target is no less than that (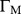 or 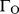) for either of the single feature tuned M and O cells; and (2) the chance 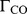 for the CO cell to be the most active neuron in response to a CO double feature target is substantial but about half of that (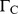 or 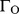) for either of the single feature tuned C and O cells. Additionally, our results show that there is no significant chance for the CM cells to be the most active neuron in response to a CM double feature target, suggesting an absence of such neurons in V1, consistent with the previous finding [10] and physiological observations [24].

The impact of the conjunctive cells on the double feature targets predicts that these cells tend to respond to their preferred stimulus more vigorously and experience weaker contextual suppressions when the contextual inputs differ from their preferred stimulus in both, rather than one, feature dimensions. This should be caused by both of the following. One is a sufficient feature tuning of the conjunctive cells in both feature dimensions, and the other is a sufficient feature tuning of the intra-cortical interactions between these cells (or between these cells and the single feature tuned cells). The roles of these two types of feature tunings in saliency are further elaborated next.

### Two types of V1 feature tuning properties

V1 saliency hypothesis implies that the highest responses to the feature singletons are higher than those to the uniformly featured non-targets. Mechanistically, this requires the following two components. First, neurons responding to the non-targets should suppress each other by iso-feature suppression, the V1 property that nearby neurons preferring the same or similar feature(s) suppress each other [41], [42], [43]. Second, the neuron preferring and responding to the target should largely escape the iso-feature suppression from neurons responding to the non-targets. These two components require two types of feature tunings to be sufficiently strong. One is the feature tuning in the input feature preference of the V1 cells. Cells preferring the target feature should prefer the non-target features much less or not at all. The other is the feature tuning of the intra-cortical interactions [5]. It specifies how quickly the intra-cortical suppression decays with the difference between the preferred features of the two interacting neurons. By sufficient feature tuning of the interactions, neurons preferring the non-targets should direct their iso-feature suppression much more to each other than to neurons preferring the target. Sufficient feature tunings in both the input preference of the neurons and interactions between neurons ensures that the neurons most activated by the target should largely escape the iso-feature suppression from the neurons responding to the non-targets.

**Figure 8:**
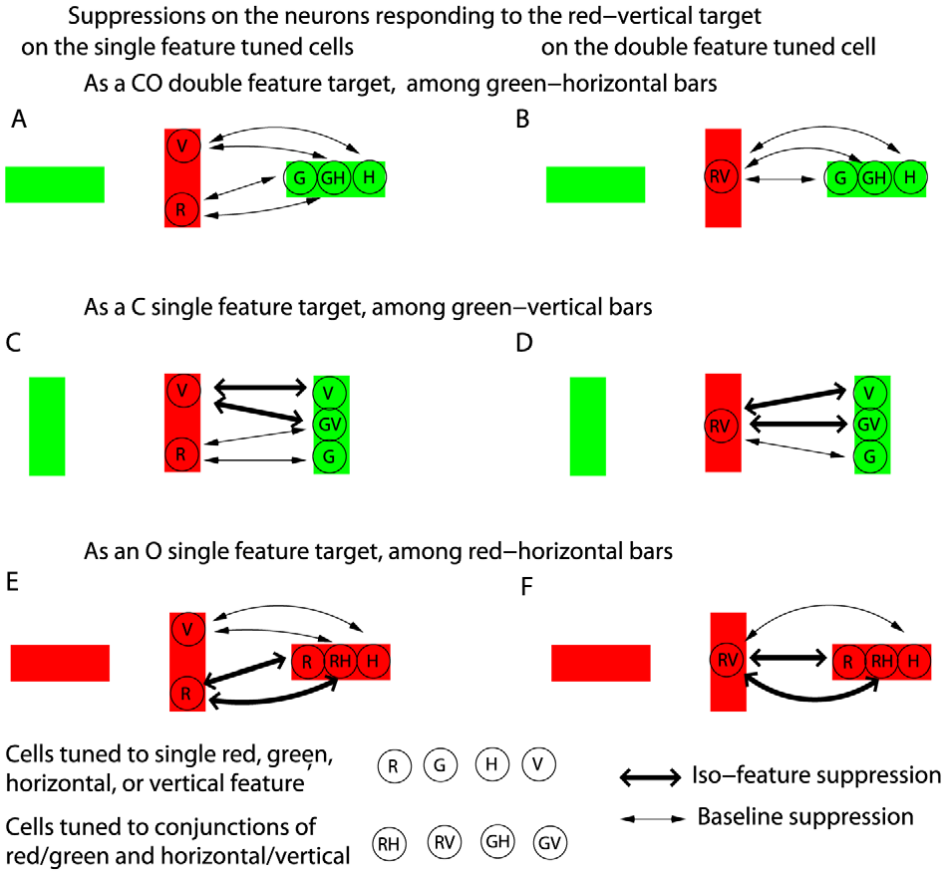
A schematic for suppression on neurons responding to the target in feature singleton search. Cases for a CO target (A and B), a C target (C and D), and a O target (E and F) are shown separately. Red and green bars are visual inputs. Circles on a bar mark neurons activated by the bar. Each neuron is marked by its preferred feature as red (R), green (G), horizontal (H), vertical (V), or a conjunction of them. Lines and curves with arrows mark (effective) suppression between two neurons, thicker for stronger suppression when the two neurons prefer the same feature. For clarity, suppression on the single feature tuned cells are shown separately (in A, C, and E) from that (in B, D, and F) on the double feature tuned cells, and interactions between the neurons responding to non-targets are not shown. Among single feature tuned neurons activated by the target, the C neuron (‘R’) is more suppressed when the target is an O target, whereas the O neuron (‘V’) is more suppressed when the target is a C target. Without the conjunctive neurons, the strongest response evoked by the CO target will be the same as the larger one of the strongest responses evoked by the C and the O targets. The CO neuron (‘RV’) responding to the target is least suppressed for the CO target (to have the redundancy gain), if suppression between conjunctive neurons is weaker when their preferred features are different in both rather than one feature dimensions (i.e., 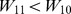 and 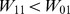, see equation (19)), or if the suppression from a single feature tuned neuron (C or O neuron) on a conjunctive neuron is weaker when they prefer different features in their shared feature dimension.

Following the analysis above, sufficient feature tuning associated with the conjunctive cells, both in input preference and in intra-cortical interactions, are required to have redundancy gains for the double feature targets. This can be understood as follows. For example, the C or O neurons, being single feature tuned, do not differentiate their responses to the target based on whether the target is a single feature target or a double feature target. Hence, the redundancy gain for the CO target requires that the CO cells respond more strongly to a double feature rather than a single feature target. To realize this, the suppression on the CO cells preferring and responding to the target from the neurons preferring and responding to the non-targets should be weaker when the target differs from the non-targets in two rather than one feature dimensions. This decreasing suppression by an increasing number of feature dimensions to distinguish the target can arise from three mechanisms, see Fig. 8. First, suppression between two CO cells is weaker when they prefer different features in both dimensions, rather than just one. Accordingly, suppression from a CO cell preferring and responding to non-targets on the CO cell preferring and responding to the target is weaker when the target is a double rather than a single feature target (compare Fig. 8B with Fig. 8DF). Second, suppression between a CO cell and a C cell is weaker when they prefer different colors. Accordingly, suppression from a C cell preferring and responding to a non-target to a CO cell preferring and responding to the target is weaker when the target and non-targets differ in color (compare Fig. 8BD with Fig. 8F). Third, suppression between a CO cell and an O cell is weaker when they prefer different orientations. Accordingly, suppression from a O cell preferring and responding to a non-target to a CO cell preferring and responding to the target is weaker when the target and non-targets differ in orientation (compare Fig. 8BF with Fig. 8D). The first mechanism alone should be sufficient, but either the second or third mechanism alone would not be. Future experiments, especially physiological and anatomical investigations, are needed to find out which sources are actually involved. Analogous conclusions apply to the MO cells and their associated feature tunings.

### The roles of single feature and conjunctive feature tuned cells in single feature target

Our behavioral data could not reveal whether the conjunctive cells are more active than the single feature tuned cells in response to the single feature targets to dictate their saliency at least occasionally. For example, the 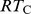 for the C target does not reveal whether a C cell or a CO cell is responsible. After all, the value of saliency is feature blind, signaled by the firing rate of the most activated V1 neuron regardless of its preferred feature(s) [6]. Our analysis has for simplicity regarded the single feature tuned cells alone as the dictating neurons for the saliencies of the single feature targets, even though the dictating responses could be from double feature tuned cells. Since these dictating responses to the single feature targets are used as the basis to calculate the contributions by the single feature tuned cells to the saliency of a double feature target, these contributions (e.g., 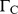 and 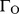) may be over-estimating the actual contributions by the single feature tuned neurons. Consequently, contributions by the double feature cells to the saliencies of the double feature targets may be under-estimated. In other words, our reported contributions 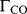 and 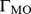 (and even 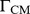) by the double feature tuned cells to the saliencies of double feature targets are in fact additional contributions by these cells beyond their hidden contributions not revealed by our RT data. These hidden contributions correspond to the contributions of the double feature tuned cells in the single feature targets. For example if the CO cells dictated the saliency of a C target in 25% of the trials and the saliency in an O target in 10% of the trials, the hidden contribution by the CO cells to the CO target could be about 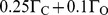 (although the actual quantity depends on more specific details), making the total contribution 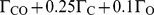 by the CO cells to the CO target. Analogous arguments apply to the contribution by the MO cells to the MO targets. Accordingly, considering that 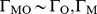, we can conclude that the dictating neuron is no less likely, and perhaps more likely, to be an MO cell than an M or an O cell.

One may ask whether the hidden contributions by the conjunctive neurons could be so much that conjunctive neurons alone dictate the saliencies of both the single and double feature targets, as if the single feature tuned neurons are invisible or absent for saliency. To answer this question, let us denote the (effective synaptic connection mediating) intra-cortical suppression between two conjunctive cells by 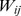, which depends on the two binary subscripts 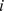 and 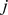 for the two feature dimensions in which the neurons are tuned. Each subscript takes value 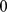 or 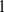 if the two neurons prefer the same or different features, respectively, in the corresponding feature dimension. The strongest suppression between the two conjunctive neurons is 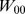, when the preferred features are the same in both feature dimensions. For example, two CO neurons suppress each other most when they prefer the same color *and* the same orientation. The second strongest level of suppression includes 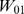 and 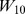, when the preferred features are different in only one feature dimension, e.g., when two CO neurons prefer different colors but the same orientation (or the same color but different orientations). For better intuition, we may refer to 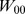 as iso-double-feature suppression and 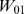 and 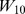 as iso-single-feature suppression (see Fig. 8). The weakest suppression is 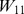, between two conjunctive neurons preferring different features in both dimensions, e.g., when the two CO neurons prefer different colors and different orientations. Feature tuning in intra-cortical suppression means that
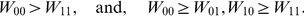
(18)Suppression 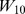 or 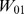 is between conjunctive neurons preferring a single feature target and those preferring the non-targets (Fig. 8DF); suppression 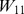 is between conjunctive neurons preferring a double feature target and those preferring the non-targets (Fig. 8B); whereas suppression 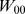 is between conjunctive neurons preferring the non-targets (not shown in Fig. 8 to avoid clutter). We have concluded above that
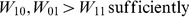
(19)helps to realize redundancy gains. Now, if conjunctive neurons alone have to dictate the saliencies of the single feature targets, then
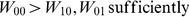
(20)is necessary to make suppression stronger on the responses to the non-targets than the target. To make all feature singletons salient and to have redundancy gains in double feature targets CO and MO but not in CM targets, no C, M, O, and CM neurons are necessary in principle, provided that equations (19) and (20) hold for both the CO and MO cells. Physiologically, there are likely a whole spectrum of single and double feature selectivities in V1 [48].

### Relationship with other studies

#### V1 physiology

The current results confirmed the previous finding by Koene and Zhaoping [10] that a statistical facilitation between the RTs for the single feature targets is sufficient to account for the shorter RTs for the CM target, but not for the CO and MO targets. Findings by both studies are consistent with physiological observations that some V1 cells are tuned conjunctively to both color and orientation [21], [22], [23], others to both orientation and motion direction [38], [39], and that few cells are tuned to both color and motion direction [24]. However, the current study differs from Koene and Zhaoping [10] in research questions asked, methodology, and outcomes. Koene and Zhaoping [10] used the behavioral RT data and the known V1 facts (the presence and absence of certain conjunctive cells) to test whether the V1 saliency hypothesis is correct, whereas the current study applies the V1 saliency hypothesis (assumed to be correct) to the behavioral data to investigate the less known V1 neural properties – the response levels and feature selectivities associated with the conjunctively cells. Koene and Zhaoping [10] found out qualitatively whether the RT redundancy gains are present in certain feature dimensions in order to determine whether the neural substrate for saliency is most likely V1 rather than the extrastriate cortices. In contrast, the current study formulates an optimization approach to predict quantitatively the probability that the conjunctive neurons should dominate the V1 responses. In addition, the current study predicts the feature tuning properties of the intra-cortical interactions associated with the conjunctive neurons. These predicted properties, in particular that the MO cells are no less likely than either of the single feature tuned neurons to dominate the responses to a MO singleton, can be experimentally tested.

Iso-feature suppression is only one of the intra-cortical interaction between V1 neurons, albeit a dominant one. Another notable interaction is colinear facilitation [49], the excitation between two V1 neurons whose preferred input bars have similar orientations and are aligned as if belonging to a single smooth curve. When a central bar (such as our visual search target) is surrounded by uniformly oriented background bars in a statistically isotropic manner, the net interaction between the (neurons responding to the) central bar and the surrounding bars is still iso-orientation suppression, stronger when the orientations of the central and surrounding bars are more similar, as observed physiologically [42]. Since the density of our non-targets is quite high, each non-target can be approximately viewed as surrounded by other non-targets isotropically and experiencing a net iso-orientation suppression as well. Hence, for our current study when it is only necessary to evaluate the net suppression on each neuron, it is not necessary to consider colinear facilitation separately.

#### The role of the extrastriate cortices for attentional guidance

There are many neurons tuned to CM conjunctions in V2 [25], [26], but few in V1 [24]. Hence, our finding of no contribution by the CM neurons provides a strong support that V1 rather than extrastriate mechanisms play the dominant role in saliency for these feature singletons. This however does not rule out the possibility that the extrastriate cortex plays a role guiding attention for other visual stimuli. Recently, depth cue was found [33] to speed up the task to locate a texture border only if this border is not salient enough for observers to report its location within one second. Since extrastriate cortices rather than V1 are thought as responsible for depth perception [27]–[32], this finding suggests that, when the target saliency is too weak, V1 signals may be insufficient to guide attention in a dominant manner. The brain areas such as the superior colliculus may coordinate and combine contributions from various cortical areas to guide attention. Superior colliculus is particularly suitable for such a role since it receives inputs from multiple brain areas including V1, extrastriate cortex, and parietal cortices, and directly controls the gaze shifts through the brain stem [50], [51]. Since longer latencies are typically required for contributions from higher brain areas, it is conceivable that the speeded or hurried decisions for attentional shifts are reached using only contributions from lower cortical areas such as V1. Meanwhile, since human gaze shifts about three times per second, and since previous works suggest that top-down factors play an increasingly dominant role to guide attention when longer latencies are allowed [4], [52], it is unclear whether the attentional guidance 800 ms after the stimulus onset could be viewed as strictly by (bottom-up) saliency alone.

#### Implications on conjunction search

**Figure 9:**
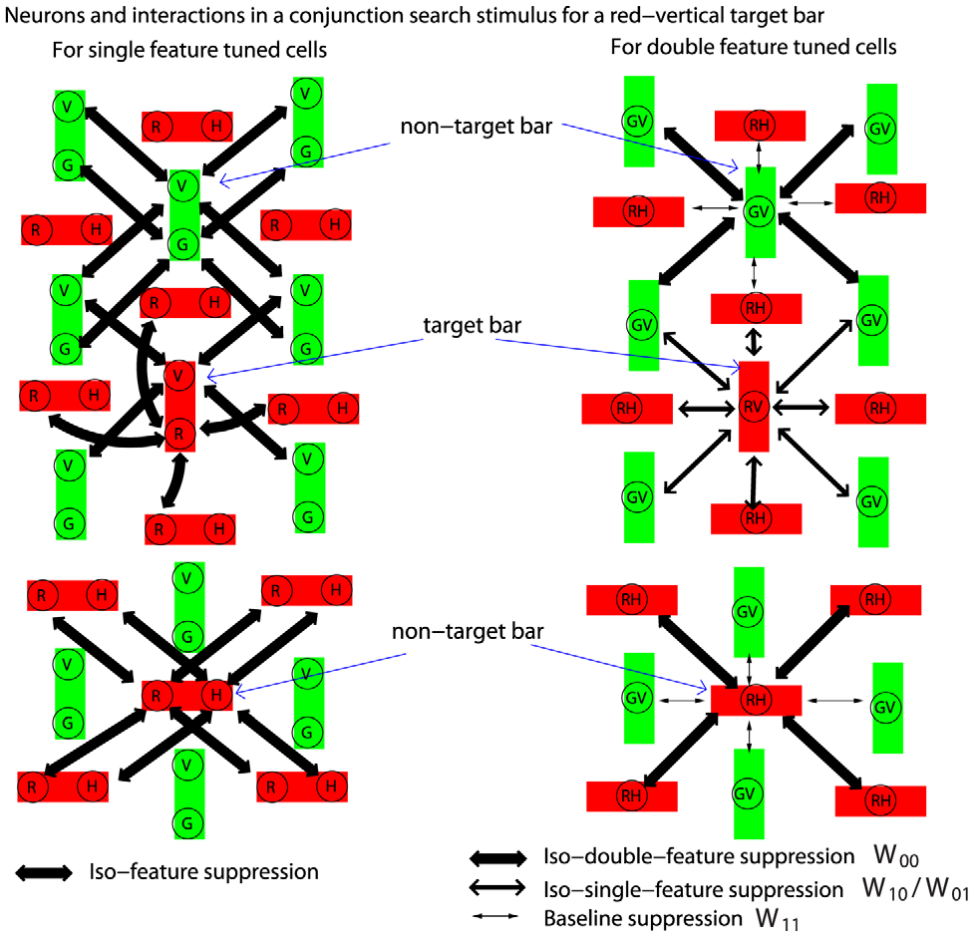
A schematic for neurons and their interactions in a conjunction search for a red-vertical target. Bars and neurons are similarly visualized as in Fig. 8. For clarity, interactions between single feature tuned neurons are shown separately (on the left) from those between conjunctive neurons. To avoid clutter, only interactions associated with neurons responding to the target bar and two of the non-target bars are shown, the baseline suppression on the single feature tuned cells and suppression between single feature tuned and conjunctive neurons are not shown. Each single feature tuned neuron, regardless of its preferred feature and regardless of whether it is responding to the target, experiences iso-feature suppression from neurons responding to about half of the neighboring bars. Hence, single feature tuned neurons cannot distinguish the target by their response levels. The conjunctive neuron (‘RV’) responding to the target experiences iso-single-feature suppression (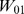 or 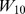) from other conjunctive neurons responding to all neighboring bars, whereas each conjunctive neuron responding to a non-target experiences iso-double-feature suppression (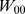) from conjunctive neurons responding to only half of the neighboring bars. The target cannot be distinguished by a higher response (from the ‘RV’ neuron) if 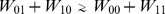, or if this neuron's response is weaker than the responses from the single feature tuned neurons.

The visual search task considered in this study is a feature search task [2], since the target can be distinguished by a unique feature, even when it is a double feature target. In contrast, when a target shares one (or more) features with some or all distractors and can only be distinguished by a particular conjunction of features, the search is much more difficult and is called a conjunction search [2]. For example, to find a red-vertical target bar among red-horizontal bars and green-vertical bars, the target conjunction is of red color feature and the vertical orientation feature, while both red and vertical features are present among the non-targets. The difficulty in conjunction searches can be easily understood if there is no conjunctive neurons. For the example above, the neurons preferring red respond to both the target and many non-targets and suppress each other by iso-color suppression; similarly, the neurons preferring vertical respond to both the target and many non-targets and suppress each other by iso-orientation suppression (see the left half of Fig. 9). Consequently, the single feature tuned neurons cannot distinguish a target by their response levels since their responses to the target are no higher statistically than their responses to the non-targets. However, one may wonder whether the conjunctive neurons preferring the unique target conjunction could distinguish the target by a relatively higher response. After all, the conjunctive neurons could respond as vigorously as the single feature tuned neurons to double feature singletons, and the conjunctive cell preferring and responding to the target may largely escape the suppression from neurons preferring and responding to the non-target conjunctions.

This question can be answered by dissecting the intra-cortical interactions associated with the conjunctive neurons only, see the right half of Fig. 9. We again use the example of the conjunctive search for red-vertical, and take for simplicity the (most difficult) situation when half of the non-targets are green-vertical and the other half are red-horizontal. Each conjunctive neuron preferring and responding to a non-target item (e.g., green-vertical) is subject to strong iso-double-feature suppression 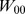 from other conjunctive neurons preferring the same color *and* the same orientation and responding to half of the non-targets in its vicinity. It should largely escape iso-feature suppression, or experience a much weaker suppression 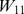, from the conjunctive neurons preferring and responding to the other half of the non-targets (e.g., red-horizontal) in the vicinity, since they prefer different color *and* different orientation. Meanwhile, a neuron preferring red-vertical and responding to the target is subject to two sources of iso-single-feature suppression: iso-color suppression 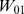 from red-horizontal preferring neurons responding to half of the non-targets in the vicinity, and iso-orientation suppression 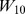 from green-vertical preferring neurons responding to the other half of the non-targets in the vicinity. Hence, the response to the target is subject to suppression 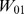 or 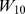 from neurons responding to all neighboring items, whereas the response to a non-target is subject to suppression 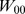 from neurons responding to only (about) half of the neighbors. Therefore, the response to the target is not distinguished unless 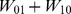 is sufficiently weaker than 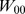 (or 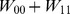 when including suppression from the conjunctive neurons preferring different features in both dimensions). For example, if two conjunctive cells do not substantially suppress each other unless they prefer the same feature in both feature dimensions, i.e., 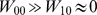, 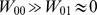, then the response to the unique target conjunction can be relatively free of suppression to make the target salient. This situation has been demonstrated in a V1 model, see Fig. 5 of Li [6].

It should be noted that the arguments above have for simplicity omitted the interactions between the single feature tuned cells and the double feature tuned cells. It is also possible that the conjunctive cell responding to the target is suppressed by the single feature tuned cells responding to the neighboring non-targets, since each non-target shares one feature in common with the target. The same conclusion in the last paragraph could still be reached if the iso-single-feature suppression (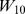 and 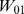) between two conjunctive cells is replaced by the iso-feature suppression between a conjunctive neuron and a single feature tuned neuron preferring the same feature in their shared feature dimension. As far as a conjunctive neuron is concerned, the pre-synaptic source for the iso-single-feature suppression may be either the double or single feature tuned neurons, or may include both. Similarly, iso-feature suppression on single feature tuned cells could arise from both the single feature tuned and conjunctively tuned cells.

Since color-orientation conjunction search is known to be difficult [2], it suggests that iso-feature contextual suppression on a CO cell (responding to its preferred input) is substantial even when the contextual inputs is different from the preferred input in one, but not both, of the two feature dimensions. This conclusion, also reached previously [10], [11], can be physiologically tested. Meanwhile, McLeod, Driver, and Crisp [53] showed that the conjunction search for a moving “X” among static “X”s and moving “O”s are relatively easy. If one treats the difference between an “X” and an “O” as a difference in orientation, this suggests that the a MO conjunction search is not too difficult, and if so, one could infer that MO neurons are not sufficiently suppressed by contextual inputs unless the contextual inputs and the preferred inputs share the same feature in both the O and M dimensions. However, a more authentic MO conjunction search is required for more confident inferences.

It is now clear that a conjunctive search should definitely be difficult if there is no V1 neurons preferring this particular conjunction. For example, there is no V1 neuron which simultaneously prefers two different orientations (or two colors) without also preferring the average orientation (or color) of the two preferred ones. Indeed, it has long been known that a unique conjunction of two different features within a single feature dimension, e.g., a conjunction of two orientations, is very difficult to find [54]. Similarly, redundancy gains involving two features in the same dimension should be absent, consistent with behavioral data [11].

#### Redundancy gains for saliency versus feature integration for object recognition

It takes longer to identify both features, color and orientation, of an object than it is to identify just one feature [55]. This is in contrast to the shorter reaction times to find a feature singleton unique in two, rather than one, feature dimensions in visual search. These two situations involve two different tasks, one is object recognition or identification and the other is feature detection or localization. These two tasks are often called the “what” and “where” tasks, respectively, and are believed to involve separate brain regions [56]. By the psychological Feature Integration Theory [2], additional processing is needed to bind two features of a single object to identify the object after the location of the object is selected by spatial attention. Meanwhile, the feature singleton detection in our task mainly involves bottom-up saliency to select the most salient location without identifying the features or objects. Indeed, observers for the task typically did not pay attention to which features distinguish the target when they pressed the button to report its location [10]. A separation between the “where” and “what” task is one of the foundations of the theoretical framework that V1 mechanisms serve the functional role of visual segmentation without classification [40], which means to segment an image region (by highlighting its boundaries with higher V1 responses) without recognizing the region. This theoretical framework has in turn inspired the V1 saliency hypothesis, which uses V1 activities to represent saliencies before decoding the visual input feature values from the very same activities [6]. Accordingly, the V1 neural activities are universal currencies for saliency regardless of their feature preferences [57].

### Concluding remarks

The V1 saliency hypothesis enables us to probe the properties of V1 neurons and intra-cortical interactions from behavioral data on visual search tasks, rather than by physiological experiments. Inferring coarse scale brain substrates from behavior is quite common in psychological studies. For example, damage to hippocampus could be inferred if somebody has difficulty forming new memories, applying the knowledge that hippocampus is a substrate for memory consolidation [58]. However, inferring neuronal level details from behavior is much less common. Many of the previous works linking physiology and behavior are to explain behavior from physiology. For example, sensory discrimination thresholds can be derived from feature tuning of the neurons and the densities of neurons involved [59]–[62]. Works to infer physiology from behavior are mainly those to infer the underlying neural channels of signal representation via sensory adaptation [63]. Behind these works are theories of optimal sensory decoding or assumptions linking neural sensitivities to behavioral sensitivities. The current work adds the V1 saliency hypothesis to the theoretical bases that can be used to link physiology to behavior, thereby extending the realms of neural mechanisms that can be probed from behavior.
